# The potential of digital molecular diagnostics for infectious diseases in sub-Saharan Africa

**DOI:** 10.1371/journal.pdig.0000064

**Published:** 2022-06-30

**Authors:** The Digital Diagnostics for Africa Network

**Affiliations:** University of British Columbia, CANADA

## Abstract

There is a large gap between diagnostic needs and diagnostic access across much of sub-Saharan Africa (SSA), particularly for infectious diseases that inflict a substantial burden of morbidity and mortality. Accurate diagnostics are essential for the correct treatment of individuals and provide vital information underpinning disease surveillance, prevention, and control strategies. Digital molecular diagnostics combine the high sensitivity and specificity of molecular detection with point-of-care format and mobile connectivity. Recent developments in these technologies create an opportunity for a radical transformation of the diagnostic ecosystem. Rather than trying to emulate diagnostic laboratory models in resource-rich settings, African countries have the potential to pioneer new models of healthcare designed around digital diagnostics. This article describes the need for new diagnostic approaches, highlights advances in digital molecular diagnostic technology, and outlines their potential for tackling infectious diseases in SSA. It then addresses the steps that will be necessary for the development and implementation of digital molecular diagnostics. Although the focus is on infectious diseases in SSA, many of the principles apply to other resource-limited settings and to noncommunicable diseases.

## Author summary

Diagnostic tests are fundamental to the practice of modern medicine, underpinning correct diagnosis and treatment. There is a global disparity in access to diagnostic tests, and much of the population of sub-Saharan Africa (SSA) does not have access to essential tests. Consequently, there is also limited data on the true burden of many diseases in SSA. Diagnostic laboratories are expensive and complex to build, run and maintain, and so increasing the number of laboratories may not be the best solution. We have identified new digital diagnostic technologies as an alternative approach with potential to bring the laboratory to the patient, wherever they may be. These technologies could transform healthcare in SSA, particularly for infectious diseases. Conducting diagnostic tests in a handheld device, often on the surface of a microchip, then may provide the accuracy of tests in a large laboratory, but in a rapid, cheap, and portable format. By transmitting real-time data, digital diagnostics also have the potential to transform surveillance of infectious diseases. This will allow prevention and control measures to be targeted where they are most needed.

## Introduction

**Fig 1:**
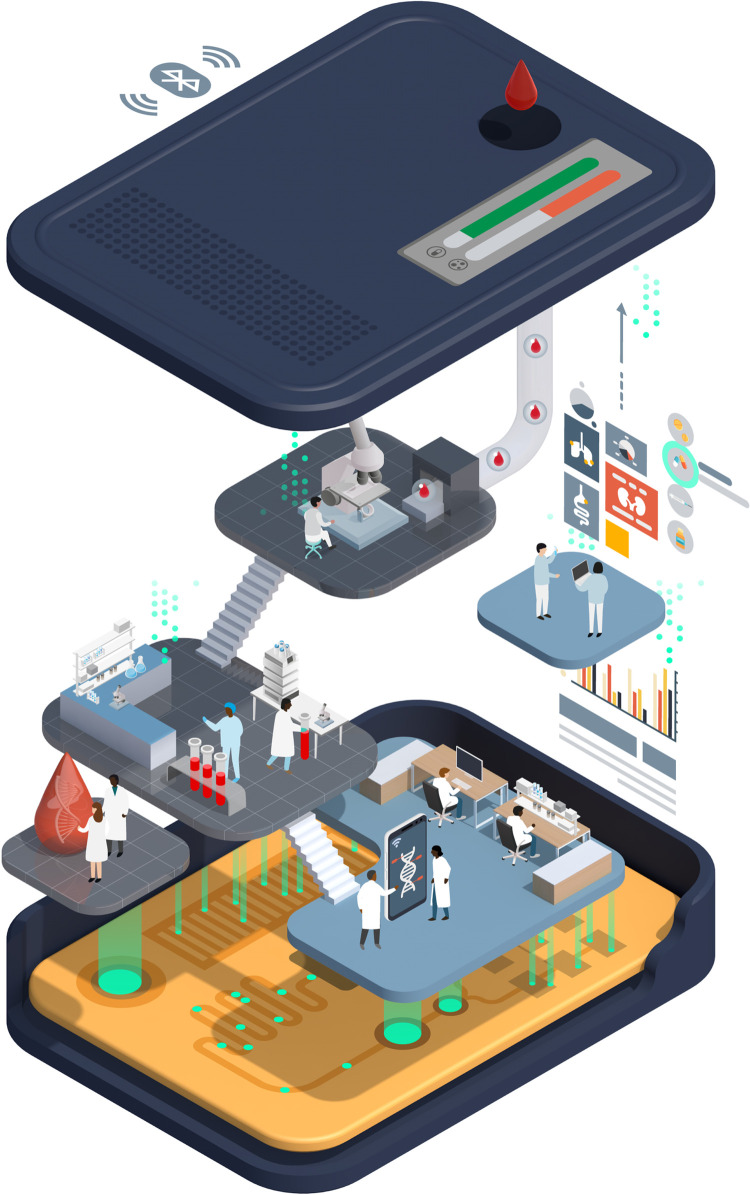
What is a digital molecular diagnostic? Throughout this article, the term “digital molecular diagnostic” describes a small electronic device, providing a sample-to-answer solution to a diagnostic problem, in a portable, easy-to-use, robust, and cheap format. Any processing of a biological sample would ideally be integrated into the device, before allowing quantitative detection of the molecules used to make the diagnosis. The molecules detected are typically nucleic acids (DNA or RNA), but could also include proteins, or small chemical molecules. Such digital diagnostics will often use lab-on-chip technology, with their defining features being the generation, processing, and storage of data. Signals from the detection of molecules undergo processing within the device, so that actionable results are reported to the user without the need for further analysis. Results may be displayed on the device itself, or linked to other interfaces such as smartphones, and decision support may be integrated. Quantitative data generated by the device can be easily and immediately transmitted to facilitate patient care and contribute to disease surveillance.

Sub-Saharan Africa (SSA) experiences the greatest gap between health needs and healthcare provision [1]. At least 50% of the population do not have access to essential health services [2]. One critical gap is easy access to accurate diagnostics [3], which is fundamental for achieving Universal Health Coverage (UHC) [4,5]. Accurate diagnostics help to ensure that correct treatments are prescribed for individuals and provide vital epidemiological information that underpins disease prevention and control strategies [6,7]. Advances in digital molecular diagnostics (defined in Fig 1) have the potential to accelerate healthcare provision towards UHC, bringing high-quality diagnostics and decision support tools to the point of care while simultaneously collecting real-time data to underpin efficient and effective disease control.

The United Nations Sustainable Development Goal 3 (SDG3) (https://sdgs.un.org/goals/goal3) sets ambitious targets for 2030, including an end to preventable deaths of newborns and children under 5 years (the group most at risk of death from infection), and epidemics of AIDS, tuberculosis, malaria, and neglected tropical diseases. These goals will only be possible through dramatic changes in access to diagnostics and treatment, along with better acquisition and use of data to efficiently target and monitor interventions. The Coronavirus Disease 2019 (COVID-19) pandemic has threatened to reverse progress already made towards achieving SDG3 in SSA [8] and highlighted the importance of diagnostics for controlling infectious diseases [9]. Improved diagnostics are therefore central to international strategies against high-burden infectious diseases [10,11], to address new pandemic threats [9,12,13] and ultimately prevent avoidable deaths. The World Bank and African Union predict a digital transformation that will accelerate trajectories of economic growth and innovation in Africa over the next decade [14]. There is an opportunity for a parallel digital revolution in diagnostics. Similar to the way that mobile phone technology has leapfrogged conventional landline infrastructure in most of SSA [15], a digital diagnostic ecosystem has the potential to replace many of the needs for conventional diagnostic laboratory infrastructure.

This article reviews the need for improved diagnostics and disease surveillance in SSA, how digital diagnostics could meet this need, recent advances and future developments in digital diagnostic technology, and approaches for successful implementation. The focus is primarily on digital molecular diagnostics for infectious diseases in SSA, but similar principles apply to digital diagnostics for noncommunicable diseases and other resource-limited settings.

### Challenges and opportunities for diagnostic ecosystems in SSA

There is enormous variation in the availability and accessibility of diagnostics within SSA, largely determined by socioeconomic and geopolitical factors [1]. State-of-the-art diagnostic facilities are available to an affluent minority in some countries, while the majority can only access or afford a small range of diagnostic tests offered through community or primary healthcare facilities [3]. WHO lists 32 essential in vitro diagnostic tests for use in community and health settings without laboratories, including dipstick tests, rapid diagnostic tests (RDTs) using lateral flow formats (e.g., malaria RDTs), and small handheld analysers, most of which can only detect single analyte [16]. However, even these remain unavailable in many settings across SSA [3,17,18]. WHO recommends additional diagnostics for healthcare facilities with clinical laboratories including microscopy, automated bench-top analysers, and nucleic acid amplification tests, many of which require skilled operators, quality control, uninterrupted electricity supplies, and supply and maintenance infrastructure. In reality, many of these tests are also unavailable, only intermittently available [19,20], or they may be prohibitively expensive, favouring use of cheaper alternatives with limited accuracy and quality assurance [21].

**Fig 2:**
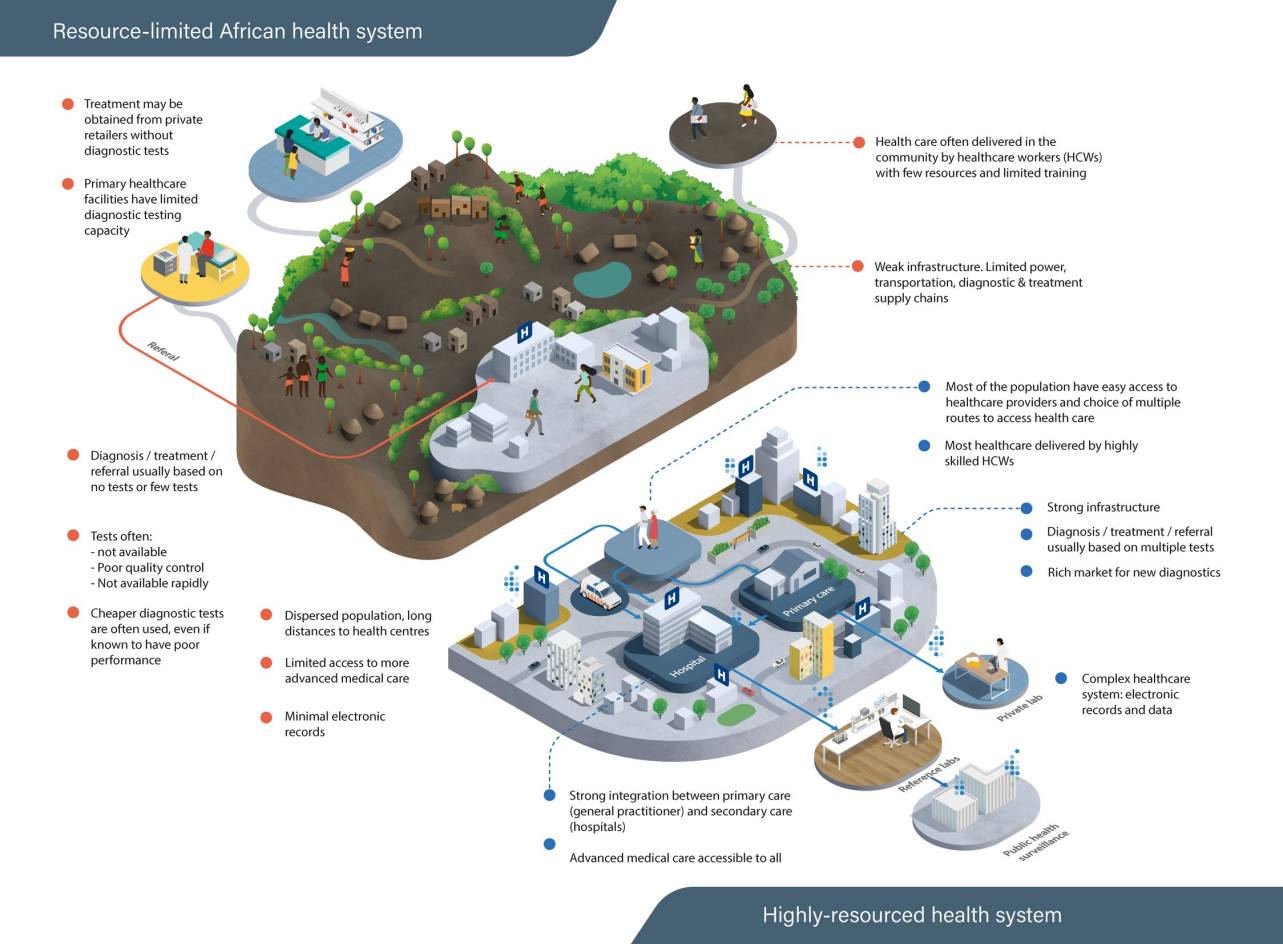
Contrasting access to healthcare and diagnostics between low-resourced SSA settings and highly resourced healthcare settings. In countries with highly resourced health systems, most of the population have easy access to health care services, often through multiple different routes. A wide range of diagnostic tests can be accessed through most healthcare providers, even if the samples need to be sent elsewhere for analysis. Strong infrastructure allows rapid transport, testing, and feedback of results, and diagnostic information can be shared between providers and patients with relative ease. Healthcare providers are often highly skilled and able to interpret the results of many different tests. In contrast, access to healthcare facilities and skilled healthcare workers in SSA is more heterogeneous and often limited, sometimes involving long journeys or incurring high costs to patients and their families. In rural and remote areas, the only accessible healthcare may be delivered by less skilled community healthcare workers, equipped with a limited range of point-of-care diagnostic tests. Healthcare facilities with high-quality laboratories do exist, but their capacity and the infrastructure to transport samples from distant facilities to these laboratories and return results in a timely fashion is often insufficient for the needs of the population, and results in further gaps in their linkage to appropriate and timely patient care. SSA, sub-Saharan Africa.

In contrast to the situation in SSA, access to a huge range of diagnostic tests is the norm in high-income countries (Fig 2). In high-resource health systems, there is often a choice of public and private healthcare providers, a variety of locations in which tests may be performed, and robust physical and digital infrastructure to transfer diagnostic samples and results between facilities, practitioners and patients. Indeed, the relative ease of diagnostic testing probably encourages overuse and overreliance on diagnostic tests [22].

A germane question is whether SSA health systems should aim to recapitulate the diagnostic ecosystems that have developed in highly resourced countries, or whether they should take an alternative path. Achieving universal access to high-quality laboratory-based diagnostics for all in SSA by 2030 seems unrealistic given the required infrastructural changes. Alternative diagnostic strategies may enable countries in SSA to “leapfrog” over the need to mimic the complex diagnostic ecosystems established in resource-rich countries [23] and lead to more efficient and economical models of healthcare, bringing high-quality diagnostics to more of the population.

### The need for new diagnostics for infectious diseases in SSA

**Fig 3:**
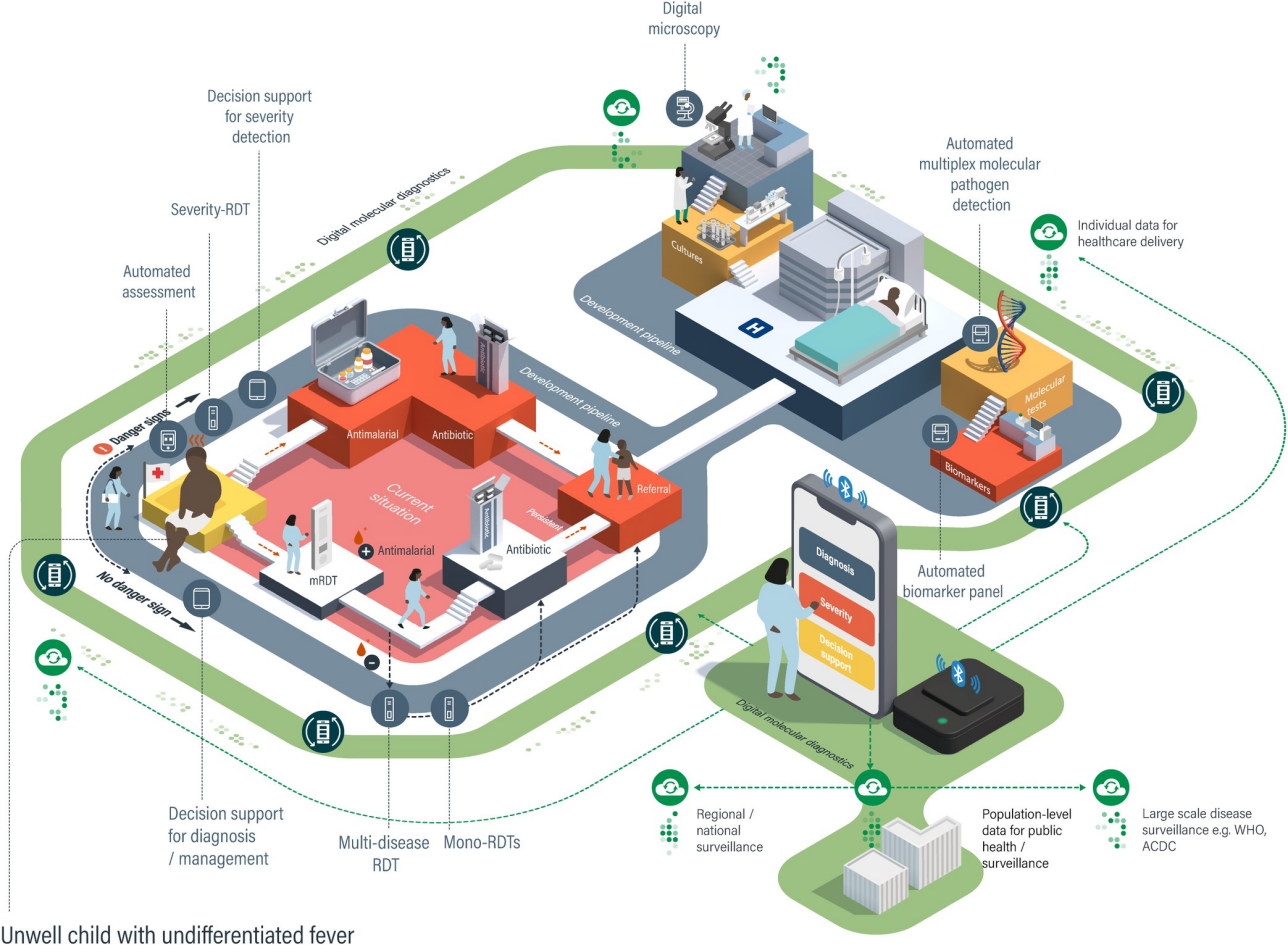
Current and future diagnostics in the integrated management of childhood febrile illness. One of the most common and important diagnostic challenges in SSA is the management of fever in young children. WHO recommends that primary healthcare workers in resource-limited settings use a syndromic approach for managing childhood febrile illness, incorporating a mRDT in malaria endemic countries (current situation, pink area). Initial management involves a triage step to establish if the child is seriously ill, based on clinical danger signs; if these are present, the child is given antimalarial treatment, antibiotics, and referred urgently to a facility where additional diagnostic tests and treatments are available. If a child is not seriously ill, then a mRDT is performed and, if positive, the child is treated with antimalarials. If the mRDT is negative, the child is evaluated for clinical signs indicating a bacterial infection (there are currently no RDTs to confirm this at the point of care) and receives antibiotics if these are present. If symptoms are persistent, then the child is referred to a higher-level facility for further assessment. Many new diagnostics and decision support tools are currently being developed to improve outcomes by addressing weaknesses at each stage in this process (grey track). Additional diagnostics are in development to improve the speed or accuracy of diagnosis in the referral healthcare facilities with clinical laboratories. New digital molecular diagnostic devices (green track) have the potential to integrate accurate diagnosis, evaluation of severity, and decision support in a single device and, through modular design of diagnostic cartridges, could provide solutions throughout the patient journey. Connectivity means that data can be shared between facilities to support patient care and for public health decision-making. mRDT, malaria rapid diagnostic test; RDT, rapid diagnostic test.

Infectious diseases are a dominant cause of premature death, chronic illness, and loss of productivity in SSA [24] and a major impediment to economic growth, education, and human development [24]. Diagnostic tests are essential for the accurate detection and optimal management of patients with infectious diseases [7], and the majority of the 122 tests recommended in WHO’s List of Essential In Vitro Diagnostics relate to infections [16]. The spectrum of human pathogens in SSA is vast, with high burdens of bacterial, viral, parasitic, and fungal diseases [25] and frequent coinfections [26]. Most infectious diseases in SSA are treated outside conventional health facilities, in the absence of clinical diagnostic laboratories, using syndromic approaches guided by few, if any, diagnostic tests [27] (Fig 3). Syndromic approaches have poor specificity, resulting in overtreatment with antimicrobials, but may also have limited sensitivity, missing cases of treatable illness [28].

Diagnosis of infectious diseases is inherently difficult because many pathogens can cause similar illness syndromes. Pathogens may not be detectable in easily sampled specimens like blood and upper respiratory tract swabs, and many organisms with pathogenic potential can be detected in nonsterile body sites without causing disease. Even when state-of-the-art diagnostic tests are performed, the causes of severe infection syndromes like sepsis or severe pneumonia cannot be microbiologically confirmed in 50% or more of cases [29]. Thus, there is an imperative to improve both the sensitivity and specificity of diagnosis at the point of care, to better identify those who will benefit most from available treatments, and to identify those at greatest risk of deterioration (who need to be transferred to a health facility that can provide more supportive treatment).

Cheap and easy-to-use lateral flow RDTs have transformed diagnosis of some infectious diseases at the point of care [30]. Malaria RDTs are a particularly good example [31]; however, they still have many limitations: They are less sensitive than expert microscopy or PCR, particularly for low parasite densities in asymptomatically infected individuals; emerging genetic mutations can render parasites undetectable by malaria RDTs [32]; they cannot identify antimalarial drug resistance; and they remain positive for weeks after successful treatment. Other RDTs feature heavily in WHO essential diagnostics list, and RDTs are in development for many of SSA’s Neglected Tropical Diseases [33]. However, transformative RDTs have not yet emerged for detection of bacterial infections in SSA, and there is still a heavy reliance on syndromic approaches in the community (Fig 3) and culture-based techniques in facilities with laboratory infrastructure. While culture is currently the “gold standard” for diagnosis of many bacterial infections, it is far from perfect because sensitivity is dramatically reduced by pretreatment with antibiotics, it is slow, and it relies on the bacteria being present in the sample that is cultured. Molecular pathogen detection, using nucleic acid amplification tests, is increasingly seen as a solution. However, this usually requires advanced laboratory infrastructure, is restricted to a predefined panel of pathogens, and rarely provides information about antimicrobial susceptibility. Nevertheless, there are examples of successful combined molecular pathogen and resistance testing, such as the Cepheid GeneXpert platform for tuberculosis [34] and the Biofire FilmArray Blood Culture Identification panel [35], which have improved the sensitivity and speed of diagnosis.

Quantification of the host response to infection is often used as a complementary approach to pathogen detection, because different pathogens elicit different immune and inflammatory responses. C-reactive protein (CRP) and procalcitonin, which are typically more elevated in blood during bacterial than viral infections, have been incorporated into RDTs [36,37]. However, it is difficult to define universal cutoffs for bacterial infection, particularly in malaria-endemic settings in SSA [38,39], where malaria can also cause an intense inflammatory response. Recently, multianalyte protein biomarker panels have been developed to increase diagnostic accuracy for bacterial infection [40], and there is promising evidence that patterns of host RNA expression in blood can distinguish between different causes of infection with high accuracy [41].

Accurate, granular and timely data on disease detection are increasingly recognised as essential to achieve the aims of SDG3 [42,43]. Such epidemiological data can be used to target interventions where they are most needed, to develop long-term policies, and also to identify emerging infectious disease threats [44]. Results of most essential infectious disease diagnostics currently used in Africa cannot be compiled in an automated, standardised, and interoperable fashion [45]. For example, malaria RDT or microscopy results may only be recorded in paper notebooks, limiting the speed and accuracy of information transfer from detection to reporting [46]. Linking clinical diagnosis to surveillance is increasingly recognised as a priority to improve disease control and elimination [42,47].

The gaps and limitations of current diagnostics and the systems that rely on them have stimulated intense innovation [11,48] (Fig 3 and Table 1). In 2003, WHO developed the Affordable, Sensitive, Specific, User-friendly, Rapid and robust, Equipment-free and Deliverable to end-users (ASSURED) criteria to guide the development of new diagnostics [49]. However, there has been no overall coordination of new diagnostic development [50], resulting in a proliferation of different tests being developed for different pathogens and different healthcare settings, potentially creating an emerging integration challenge. Furthermore, the ASSURED criteria, and most target product profiles (TPPs) for individual tests, do not specify the need for an intrinsic link between diagnosis and surveillance of infection [51], resulting in a recent call for new “REASSURED” criteria, including real-time connectivity of data in future specifications [13].

**Table 1:** Translation of molecular detection towards point-of-care digital diagnostics.

Type of molecule detected	Examples of current technology	Limitations of current technology	Selected benchtop digital platforms in development but not yet in widespread use in Africa			
			Digital platform (manufacturer)	Targets	Key characteristics	Prospects for portable point-of-care digital diagnostics
Nucleic acid: • *DNA* • *RNA*	Nested PCR qPCR rt-PCR LAMP RT-LAMP Paper-based PCR NGS	Most require skilled operators Expensive instrumentation Require secure power supply Time consuming Long turnaround time Paper-based PCR is affected by working conditions like pH and temperature NGS requires sophisticated infrastructure	HostDx Fever (*Inflammatix*)	Host response RNA profiles	PCR based - Sample to answer on a solid-state heating/cooling, extraction and amplification platform - Rapid analyte quantification without need for standard curve	Possible, but requires miniaturisation of RNA detection and computational processes into a handheld format
			LabDisk (*SpinDiag*)	DNA markers of AMR - MRSA, VRE, ESBLs, Carbapenem resistance	Nested PCR Based on centrifugally operated microfluidics on a disposable cartridge, allowing for a rapid sample to answer PCR test - Potential for software to support algorithm-based decision - Easy data interpretation	Centrifugal process will be challenging to miniaturise into a handheld device
			BLINK ONE (*BLINK*)	DNA/RNA Protein Cells	PCR based Fully integrated on cartridge and encoded with reactor beads for amplification of different analytes to produce highly multiplexed molecular assays with single molecule sensitivity	Instrument footprint is suitable for health centres with laboratory facilities and may not be easily miniaturised into handheld test devices
Protein: • *Pathogen antigens* • *Antibodies* • *Host inflammatory response proteins*	Immunoassays* Immunohistochemistry Bioassays* Lateral flow assays* Agglutination tests	Variable analytical performance Sometimes complex sample processing Sometimes time consuming	OJ-Bio	HIV biomarkers (anti-gp41 and anti-p24)	Digital platform based on SH-SAW biosensors which uses microelectronic components to detect HIV antibodies without any need for multistep washing or component labelling. Smartphone and wireless connectivity with facile electronic data capture	A good model for miniaturised digital diagnostic devices, which successfully combines rapid biomarker detection and electronic data sharing. The capability of geo-location is beneficial for faster access to therapy for persons who test positive or counselling for those who test negative
			abioSCOPE nanofluidic immunoassay technology (Abionic)	- Sepsis risk markers - Emerging virus panel - GI panel - STI panel - AMR panel	Immunoassays Nanofluidic technology with laser detection/quantification of immunocomplexes	Laser detection may be difficult to integrate into a portable device; however, the analysers allow real-time data connectivity (by encrypted data transfer) to external data management system by barcode scanning
			Spinit centrifugal microfluidic platform (biosurfit)	- CRP - HbA1c	Immunoassays performed with surface plasmon resonance using a polarised laser beam; Clinical chemistry measuring absorbance at multiple wavelengths by means of LEDs Haematology via an integrated Microscope and standard dyes - Fully integrated on cartridge - Rapid turnaround time (4 to 12 minutes)	Current architecture is unsuitable for miniaturisation into handheld POCT
			ImmunoPoc/ImmunoXpert (*MeMed*)	Host immune signatures (CRP, TRAIL IP-10)	Immunoassays ELISA-based assay with superior accuracy (compared to clinical parameters) in differentiating bacterial and viral infections	Suitable for miniaturisation into portable immunocomplex detection tests with the possibility to integrate provide real-time data connectivity
			DPP Fever panel (*Chembio Diagnostics*)	Malaria, Dengue, Ebola, Lassa, Marburg, Chikungunya Syphilis, HIV, and SARS-CoV-2	Immunoassays Cost-effective chromatographic immunoassay technology with a digital reader that reports a test electronically enhanced multiplex capability up to 8 biomarkers	Handheld analysers and small sample volumes are suitable for POCT in LMICs. Upgraded accessory smart digital readers can provide real-time connectivity and enhance use experience
Small molecules: • *Drugs* • *Toxins* • *Chemicals*	Blood biochemistry* Clinical chemistry* Spectroscopy Chromatography Colorimetry; Mass spectrometry	Variable analytical performance Complex sample processing Expensive instrumentation Require skilled operators	Evidence MultiSTAT (*Randox*)	- Drugs - Drug metabolites - Single molecule biomarkers	Immunoassays - Combines a biochip array technology with chemiluminescence - Wide range of forensic matrices - Multiplexing (up to 44 analytes) - Short turnaround time (17 minutes) - Sample processing not required	Potentially suitable for miniaturisation into lab-on-chip digital diagnostics
			FINDER digital microfluidics platform (*Baebies*)	- Total serum bilirubin - Albumin - G6PD	Biochemical, enzymatic, and immunoassays - Based on digital microfluidics fully integrated in a disposable cartridge - Low sample and reagent volumes - Multiplexing	Potentially suitable for miniaturisation into lab-on-chip digital diagnostics

*Relatively widespread in African laboratories.

AMR, antimicrobial resistance; CRP, C-reactive protein; ESBL, Extended Spectrum Beta Lactamases; G6PD, glucose-6-phosphate dehydrogenase; GI, gastrointestinal; HbA1c, glycated haemoglobin; IP-10, interferon gamma induced protein-10; LAMP, loop-mediated isothermal amplification; LED, light-emitting diode; LMIC, low- and middle-income country; MRSA, Methicillin-resistant Staphylococcus aureus; NGS, next generation sequencing; PCR, polymerase chain reaction; POCT, point-of-care testing; qPCR, quantitative polymerase chain reaction; RT-LAMP, reverse-transcription LAMP; rt-PCR, reverse transcription PCR; SARS-CoV-2, Severe Acute Respiratory Syndrome Coronavirus 2; SH-SAW, shear horizontal surface acoustic wave; STI, sexually transmitted infection; TRAIL, TNF-related apoptosis inducing ligand; VRE, Vancomycin-Resistant Enterococcus.

### Current status and potential of digital molecular diagnostics

The feasibility of incorporating molecular assays into simple, automated systems is now well established, with many examples of small benchtop devices emerging (Table 1), but the next generation of digital molecular diagnostics will refine these approaches to meet the REASSURED criteria as truly portable point-of-care tests with real-time connectivity. Significant efforts have been directed towards the development of lab-on-chip platforms for the rapid point-of-care detection of infection, although challenges still remain. These challenges include efficient, low-cost, and rapid nucleic acid extraction; sensitive and specific detection of the target pathogen or host-response; and creating a digital record of the test. Innovative solutions have emerged using microfluidic cartridges for sample preparation and protein or nucleic acid detection [52–54], paper-based microfluidic and detection systems [55–58], and electrochemical biosensors [59–61]. Relatively few emerging digital diagnostics already include a dedicated mobile phone-based application for digital records [58,62], but this will inevitably increase as technologies advance towards clinical use.

Digital molecular diagnostics are more complex to develop and will be more expensive to produce than competitor lateral flow RDTs, but they offer numerous advantages that could compensate for their cost. Digital molecular diagnostics can simultaneously measure multiple analytes (multiplexing) with real-time transfer of fully quantitative raw and integrated data, which can be presented in a variety of formats tailored to the user. Multiplexing, to detect multiple pathogens, biomarkers, or their combinations, can readily extend the range of digital molecular diagnostic tests on small volume samples [41], using innovations in microfluidic sample processing [63], and feature extraction from the quantitative data generated during molecular detection [64]. Therefore, panels of relevant pathogen genes, biomarkers to distinguish between multiple classes of pathogen [41,65], and biomarkers of severity [66,67] could be combined in a single test. This creates the possibility of personalised treatment, for example, detecting both malaria parasites and molecular markers of resistance to common antimalarials, allowing the most effective treatment to be selected; or detecting glucose-6-phosphate dehydrogenase deficiency mutations at the same time as *Plasmodium vivax* detection, to indicate the safety of radical cure with primaquine or tafenoquine [68,69]. Whereas current lateral flow RDTs give binary (positive or negative) or, at best, qualitative (e.g., negative, equivocal, weak positive, and strong positive) results, full quantitation of analytes by digital molecular diagnostics can provide additional information on pathogen load or the extent of derangement of biomarkers, which can, in turn, indicate whether a pathogen is likely to be the cause of an illness [70], inform prognosis [71], or indicate transmissibility [72,73]. Evolving approaches to diagnosis based on integration of quantitative data from multiple analytes, such as disease risk scores using gene expression levels [65,74], could easily be implemented in digital diagnostics.

Data integration does not have to be limited to sample measurements; user-specified data such as the age of the patient (which guides normal ranges of analytes [6]), or even a clinician’s estimate of the pretest probability of a diagnosis [75], could also be incorporated. Digital diagnostics could also have tuneable characteristics to allow the same device to have multiple purposes. For example, in “screening mode,” the highest possible sensitivity would be used to detect low-level asymptomatic malaria parasite infections in a community test-and-treat programme, whereas in “clinical mode,” the same device would only report infections with a parasite density above a certain threshold associated with symptomatic illness that requires treatment (although all data would be available for export). Similarly, the results delivered by the device could be tailored to the user, with simple results and instructions for community health workers and more nuanced results with quantitative data or probabilities of diagnoses available for experienced clinicians. Integrated decision support could facilitate modular testing, where the results of one test may produce a recommendation to run further tests using different test cartridges.

Combining data integration and real-time connectivity, complete data generated by digital diagnostic devices can be exported and shared quickly enough to influence decision-making: for quality control; for logistical purposes such as matching diagnostic use with resupply of health facilities; and using geolocation data to monitor trends in diagnoses over a range of geographic scales. As data accumulate, it will become possible to use automated algorithms to detect patterns of results, identifying or predicting outbreaks, and allowing additional resources to be targeted to where they might be most needed. Eventually intelligent systems may be developed using accumulated data from patients and the environment to feedback to digital diagnostic devices, adjusting device performance to better suit the local context. Maximising the value of connectivity and data is likely to be the most important determinant of success of digital diagnostics over current RDTs.

### Considerations for bringing digital molecular diagnostics into practice

**Fig 4:**
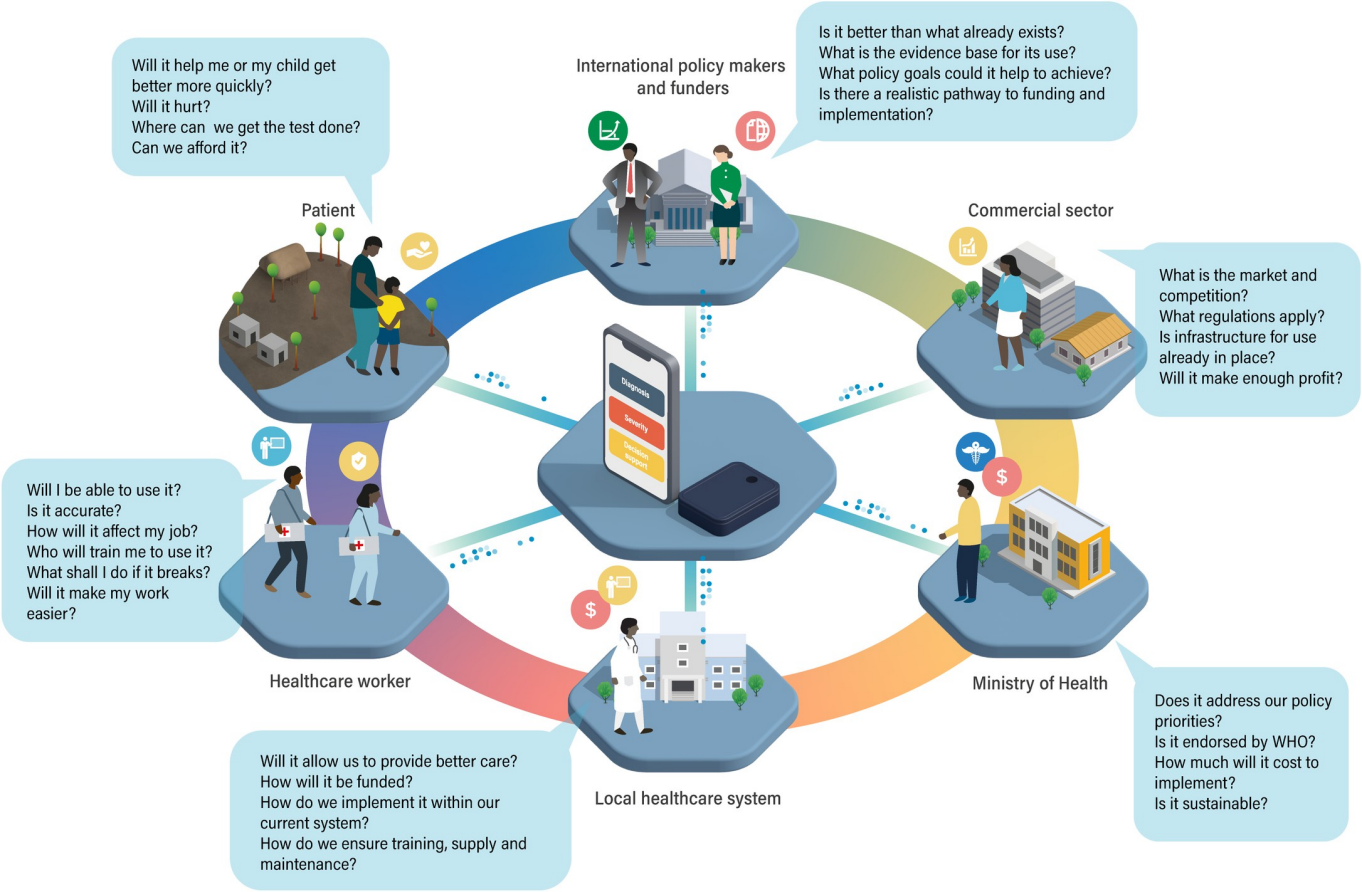
Understanding the perspective of users and stakeholders. To produce new digital diagnostics that will be widely used, it is important to understand the perspectives of all stakeholders involved in and impacted by their implementation. Understanding the perspectives of patients and healthcare workers is important, but consideration must also be given to the broader health system, government organisations, the commercial sector, international funders, and policy makers. This may start with mapping who the stakeholders are, identifying their needs, discussing their expectations for a new diagnostic, and engaging them throughout product design, development and evaluation in a codesign process.

**Fig 5:**
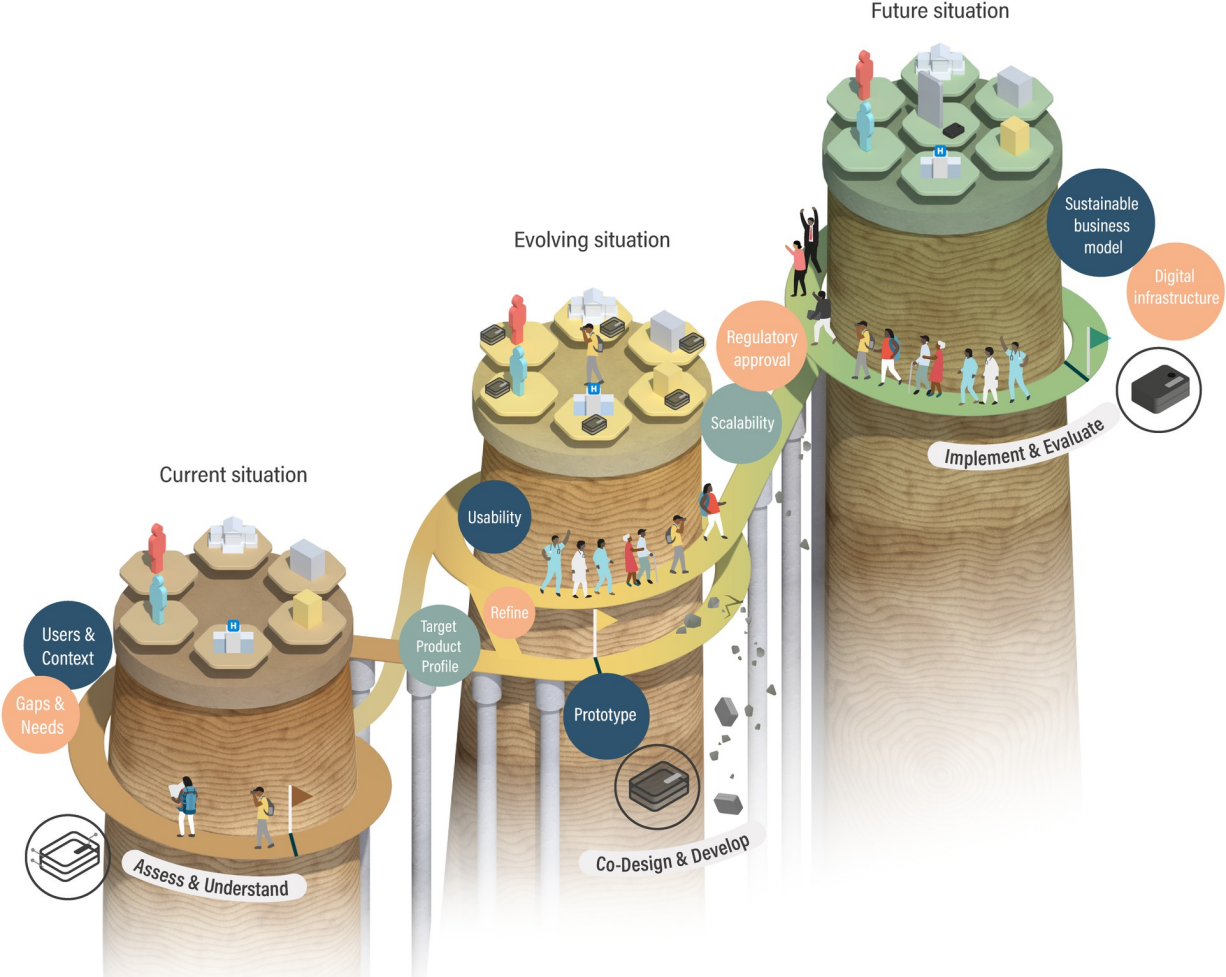
A roadmap for digital molecular diagnostic development. The development of new diagnostics is not linear, although it can be imagined as a progressive and staged process. At the outset, the current gaps and needs should be assessed and use cases developed. Context-appropriate TPPs should be developed in partnership with the potential users. Desirability (will people want to use it?), feasibility (is it technically possible?), viability (what is affordable?), and sustainability (long-term funding, readiness of and integration into the health system) should also be considered from the start of development. Prototype devices meeting the TPP are tested and refined through an iterative codevelopment and codesign process with users and an increasing number of other stakeholders who influence the diagnostic ecosystem. To bridge from prototype to implementation, scalability must be addressed, regulatory approvals gained, and continuous evaluation should ensure sustainable business models and compatibility with the evolving digital infrastructure. TPP, target product profile.

Despite their potential benefits, there are significant challenges for implementation of digital molecular diagnostics, with high risk of failure if the complexities of health systems and the diagnostic ecosystem are underestimated [76]. Some lessons can be learned from the development of emerging portable RDT readers, which provide a real-time connectivity solution for lateral flow devices [77]. The development of these devices demonstrates the importance of combining technical development with assessment of the need (use case) for new technology, and its desirability, feasibility, viability, and sustainability in the intended use settings. Other work has shown the importance of consulting and establishing partnerships with intended users and stakeholders (Fig 4) [78–80] to develop a strong value proposition for the new diagnostic. Wider technological, economic, and political constraints of the current diagnostic ecosystem and the cost–benefit ratio of new technology also need to be considered early in development (Fig 5) [81–83].

Formulating all of these considerations will allow an appropriate TPP to be established (Fig 5) [80], which specifies the characteristics required for the diagnostic device across domains from accuracy to cost, from power consumption to connectivity. TPPs for many diagnostic tests have already been developed by policy makers and international organisations, such as WHO and FIND [11,48,84], where standardised performance or alignment with policy aims is required. Many good diagnostic concepts fail because they cannot meet the TPP for their intended use [85], but meeting a prespecified TPP does not guarantee success, and should not replace the need to assess and understand the diagnostic ecosystem. Given the unique features of digital diagnostics, TPPs will need to include specifications for the data that will be generated, including measures to ensure data privacy and security [86,87]. Meeting the TPP is not the end of development, but a step to further optimisation through codesign and development with healthcare providers, service users, and policy makers, which will maximise acceptability, practicality, and, ultimately, adoption into practice (Fig 5) [78].

An iterative process of codevelopment and design can create a fit-for-purpose product, but will not guarantee widespread and sustainable implementation [88], nor the intended transformation of the diagnostic ecosystem. Beside usability, the prototype needs to be developed through levels of technology readiness [89], with rigorous testing, to ensure the TPP is achieved within the intended use environments, where accuracy, turnaround time, simplicity, portability, and cost, become increasingly important. It is important to demonstrate the diagnostic performance of the new tests in their intended use setting and also in comparison to gold standard diagnostics [90].

Crossing from development to implementation requires that digital molecular diagnostics are scalable, economically viable, have the evidence base required to achieve regulatory approval, and have endorsement at national (and often international) levels in the form of guidelines and policies [91]. A “reuse and improve” approach can facilitate this, by repurposing and enhancing existing technologies and infrastructure, which are already familiar in the use setting. This can reduce the need for training and support for the use of the new diagnostics [92], accelerate development and testing, and minimise costs [93]. Digital molecular diagnostics can capitalise on the success of mobile phones in Africa [92], to facilitate real-time connectivity and to provide a familiar user interface. Around 75% of the SSA is already covered by 3G signal, and 50% (and rapidly expanding) by 4G signal, far exceeding hard-wired connectivity [94–96]. Bringing faster, cheaper, and more efficient connectivity to remote areas is a parallel field of intense innovation (e.g., see, https://www.janga.la) and creates opportunities for partnerships that will be major enablers for the success of digital diagnostics. Furthermore, many of the mass-produced electronic components used in mobile phones can be used to build digital diagnostics, ensuring plentiful supply, preoptimisation for low power consumption, and economies of scale. Although most mobile phones are currently imported into SSA, some smartphones are now being fully manufactured in SSA, suggesting that manufacturing of digital molecular diagnostics in SSA is also a realistic aspiration [96]. Mobile phones have also become trusted financial instruments in SSA, providing simple, accessible, and cheap tools to transfer money between phone users [97,98]. Building on these principles could enable new models of funding for digital diagnostic tests where mobile payment systems can be leveraged to connect patients, healthcare providers, and healthcare payers (such as insurers and donors) [99].

Each SSA country has its own regulatory approval processes for new in vitro diagnostics, although approval is often facilitated if diagnostics have already met respected international standards and may become more streamlined with establishment of an African Medicines Agency [100]. The route to approval for pathogen detection tests is relatively straightforward, because it is possible to demonstrate analytical sensitivity and specificity on reference material and in comparison to gold standard tests. The route to approval of transformative host response based–diagnostics is less clear, especially if the intention is that the device provides both aetiological diagnosis and decision support. Evidence for regulatory approval may require large clinical Phase III trials that demonstrate both safety and efficacy of using this approach to guide patient management in situations where gold standard diagnosis is possible and also in the intended use setting where the gold standard may not be available. Dialogue with regulators to establish the likely requirements will be needed early in the development process.

Additional national and international regulations may also apply to the use and sharing of data, and regulations governing patient identifiable information will typically be separate from regulations governing in vitro diagnostics. Nevertheless, data sharing for patient benefit, and for collective exploitation between stakeholders and communities, is fundamental to improving healthcare efficiency and addressing inequalities [101]. Integration of digital diagnostics with existing open-source health information systems, such as the increasingly popular DHIS2 (https://dhis2.org), will help to resolve many regulatory and security issues, as well as facilitating scale-up. Analyses across multiple data platforms could be facilitated using a federated learning approach [102], in which analysis algorithms rather than data are transferred. This might produce, for example, granular but not precise geolocation of detected cases of a disease, such that privacy is not compromised, but interventions can be targeted at an appropriate scale.

Large-scale implementation of digital molecular diagnostics requires changes in systems and behaviour, under the influence of policy makers and funders, and with support from the commercial sector. Therefore, developers also need to consider how they can engage with these groups to guide development of a product that will align with policy and commercial sector interests and justify investments that may be required to bring new diagnostics to market. National governments often look to international organisations such as WHO and the Africa CDC for recommendations before changing diagnostic policy, and those policy changes may be easier if funding is also made available, through channels such as the Global Fund. Therefore, setting diagnostic development in the context of existing international policies and strategies, and seeking to engage with policy makers and funders, is also important. Commercial partners will be needed for sustainable implementation—potentially lucrative roles if widespread implementation is successful. However, if digital diagnostics replace other diagnostics and alter the use of other commodities in the health system, then other commercial entities risk losing business. Therefore, new digital molecular diagnostics may not be universally welcomed, and may even be obstructed, and this should be anticipated in planning for scale-up.

## Conclusions

Innovations in digital molecular diagnostic technology have set the scene for the next generation of point-of-care tests, which could catalyse transformations in healthcare delivery. SSA could reap great benefits from early adoption of digital molecular diagnostics, using them to accelerate progress towards UHC and SDG3. Despite clear potential for widespread benefits from digital molecular diagnostics, successful implementation presents major challenges that must be addressed, including readiness and willingness to change across many sectors, generating stakeholder and funder support to catalyse this change, and negotiating the practical challenges of physical, digital, and regulatory infrastructure.

## Supporting information

S1 Text **Table A:** Names and affiliations of The Digital Diagnostics for Africa Network Contributors. **Table B:** Author contributions (CRediT Taxonomy).
(DOCX)
