# Correction: The potential of digital molecular diagnostics for infectious diseases in sub-Saharan Africa

**DOI:** 10.1371/journal.pdig.0000105

**Published:** 2022-08-30

**Authors:** The PLOS Digital Health Staff

## Notice of Republication

This article was republished on August 12, 2022 to correct the XML version of the article which displayed an incorrect author list in the byline. The publisher apologizes for the errors. Please download this article again to view the correct version. The originally published, uncorrected article and the republished, corrected articles are provided here for reference.
