# Supplementary material for: The potential of digital molecular diagnostics for infectious diseases in sub-Saharan Africa
Source: PLOS Digit Health. 2022 Jun 30;1(6):e0000064. doi: 10.1371/journal.pdig.0000064 (PMC9931288; doi:10.1371/journal.pdig.0000064)
Supplement: S1 Text — Table A: Names and affiliations of The Digital Diagnostics for Africa Network Contributors. Table B: Author contributions (CRediT Taxonomy). (DOCX) [file pdig.0000064.s001.docx]

**S1 Text**

**Table A. Names and Affiliations of The Digital Diagnostics for Africa Network Contributors**

| Initials | Last Name | Affiliation |
| --- | --- | --- |
| A.A.M. | Omer | Faculty of Medicine, University of Khartoum, Khartoum, Sudan |
| A.J. | Cunnington | 1. Section of Paediatric Infectious Disease, Department of Infectious Disease, Imperial College London, UK. 2. Centre for Paediatrics and Child Health, Imperial College London, UK |
| A.O. | Mohamed | Department of Biochemistry, Faculty of Medicine, University of Khartoum, Khartoum, Sudan |
| A.W. | Sifuna | Department of Medical Biochemistry, Masinde Muliro University of Science and Technology, Kakamega, Kenya |
| C. | Bonnington | Malaria Consortium, London, United Kingdom |
| C.E. | Costelloe | Global Digital Health Unit, Department of Primary Care and Public Health, Imperial College London, London, UK |
| D.A. | Akogo | minoHealth AI Labs, Accra, Ghana |
| F.D. | Krampa | West African Centre for Cell Biology of Infectious Pathogens, University of Ghana, Accra, Ghana |
| F.M. | Piffer | Department of Life Sciences, Imperial College London, London, UK |
| G.A. | Awandare | West African Centre for Cell Biology of Infectious Pathogens, University of Ghana, Accra, Ghana |
| H. | Tinto | Institut de Recherche en Sciences de la Santé (IRSS), Clinical Research Unit of Nanoro, Nanoro, Burkina Faso |
| I. | Pennisi | Section of Paediatric Infectious Disease, Department of Infectious Disease, Imperial College London, London, UK. |
| I.A. | Samori | minoHealth AI Labs, Accra, Ghana |
| J. | Achan | Malaria Consortium Uganda, Kampala, Uganda |
| J. | Rodriguez-Manzano | Section of Adult Infectious Disease, Department of Infectious Disease, Imperial College London, London, UK |
| J. | Balen | School of Health and Related Research, University of Sheffield, Sheffield, UK |
| J. | Baum | a. Department of Life Sciences, Imperial College London, London, UK b. Institute of Infection, Imperial College London, London, UK |
| J. | Makani | Department of Haematology and Blood Transfusion, Muhimbili University of Health and Allied Sciences, Dar-es-Salaam, Tanzania |
| J.A. | Herberg | 1. Section of Paediatric Infectious Disease, Department of Infectious Disease, Imperial College London, UK. 2. Centre for Paediatrics and Child Health, Imperial College London, UK |
| K. | Malpartida-Cardenas | Centre for Bio-inspired Technology, Department of Electrical and Electronic Engineering, Imperial College London, London, UK |
| K. | Baker | Malaria Consortium, London, UK |
| L.C. | Okell | MRC Centre for Global Infectious Disease Analysis, Department of Infectious Disease Epidemiology, Imperial College London, London UK |
| L.P. | de Witte | School of Health and Related Research, University of Sheffield, Sheffield, UK |
| LE | Amoah | NMIMR, University of Ghana |
| M. | Kaforou | 1. Section of Paediatric Infectious Disease, Department of Infectious Disease, Imperial College London, UK. 2. Centre for Paediatrics and Child Health, Imperial College London, UK |
| M. | Levin | 1. Section of Paediatric Infectious Disease, Department of Infectious Disease, Imperial College London, UK. 2. Centre for Paediatrics and Child Health, Imperial College London, UK |
| M. | Mahdi | Department of Parasitology and Medical Entomology, Institute of Endemic Diseases, University of Khartoum, Khartoum, Sudan |
| N. | Moser | Centre for Bio-Inspired Technology, Department of Electrical and Electronic Engineering, Imperial College London, London, UK. |
| N. | Mursi | Department of Molecular Medicine, Institute of Endemic Disease, University of Khartoum, Khartoum, Sudan |
| P. | Georgiou | Centre for Bio-Inspired Technology, Department of Electrical and Electronic Engineering, Imperial College London, London, UK. |
| P.M | Hamade | Malaria Consortium, London, UK |
| S. | Duodu | 1. West African Centre for Cell Biology of Infectious Pathogens, University of Ghana, Accra, Ghana  2. Department of Biochemistry, Cell and Molecular Biology, University of Ghana, Accra, Ghana. |
| S. | Gamil | Department of Biochemistry, Faculty of Medicine, University of Khartoum, Khartoum, Sudan |
| S. | Yeung | Clinical Research Department, London School of Hygiene and Tropical Medicine, London, UK |
| T. | Porat | Dyson School of Design Engineering, Imperial College London, London, UK |
| U. | D'Alessandro | MRC Unit The Gambia at the London School of Hygiene and Tropical Medicine, Fajara, The Gambia |
| W. L. | Baxter | Dyson School of Design Engineering, Imperial College London, London, UK |
| X.-L. | Palmer | minoHealth AI Labs, Accra, Ghana |

**Table B. Author Contributions (CRediT Taxonomy)**

| Initials | Last Name | Conceptualization | Data  Curation | Formal  Analysis | Funding Acquisition | Investigation | Methodology | Project Administration | Resources | Software | Supervision | Validation | Visualization | Writing –  Original Draft | Writing –  Review & Editing |
| --- | --- | --- | --- | --- | --- | --- | --- | --- | --- | --- | --- | --- | --- | --- | --- |
| A.J. | **Cunnington** |  |  |  |  |  |  |  |  |  |  |  |  |  |  |
| A.A.M. | **Omer** |  |  |  |  |  |  |  |  |  |  |  |  |  |  |
| A.O. | **Mohamed** |  |  |  |  |  |  |  |  |  |  |  |  |  |  |
| AW | **Sifuna** |  |  |  |  |  |  |  |  |  |  |  |  |  |  |
| C. | **Bonnington** |  |  |  |  |  |  |  |  |  |  |  |  |  |  |
| CE | **Costelloe** |  |  |  |  |  |  |  |  |  |  |  |  |  |  |
| D.A. | **Akogo** |  |  |  |  |  |  |  |  |  |  |  |  |  |  |
| F.D. | **Krampa** |  |  |  |  |  |  |  |  |  |  |  |  |  |  |
| F.M. | **Piffer** |  |  |  |  |  |  |  |  |  |  |  |  |  |  |
| G.A. | **Awandare** |  |  |  |  |  |  |  |  |  |  |  |  |  |  |
| H. | **Tinto** |  |  |  |  |  |  |  |  |  |  |  |  |  |  |
| I.A. | **Samori** |  |  |  |  |  |  |  |  |  |  |  |  |  |  |
| I. | **Pennisi** |  |  |  |  |  |  |  |  |  |  |  |  |  |  |
| J | **Achan** |  |  |  |  |  |  |  |  |  |  |  |  |  |  |
| J.A. | **Herberg** |  |  |  |  |  |  |  |  |  |  |  |  |  |  |
| J. | **Balen** |  |  |  |  |  |  |  |  |  |  |  |  |  |  |
| J. | **Baum** |  |  |  |  |  |  |  |  |  |  |  |  |  |  |
| J. | **Makani** |  |  |  |  |  |  |  |  |  |  |  |  |  |  |
| J. | **Rodriguez-Manzano** |  |  |  |  |  |  |  |  |  |  |  |  |  |  |
| K. | **Baker** |  |  |  |  |  |  |  |  |  |  |  |  |  |  |
| K. | **Malpartida-Cardenas** |  |  |  |  |  |  |  |  |  |  |  |  |  |  |
| L.E. | **Amoah** |  |  |  |  |  |  |  |  |  |  |  |  |  |  |
| L.C. | **Okell** |  |  |  |  |  |  |  |  |  |  |  |  |  |  |
| L.P. | **de Witte** |  |  |  |  |  |  |  |  |  |  |  |  |  |  |
| M. | **Kaforou** |  |  |  |  |  |  |  |  |  |  |  |  |  |  |
| M. | **Levin** |  |  |  |  |  |  |  |  |  |  |  |  |  |  |
| M. | **Mahdi** |  |  |  |  |  |  |  |  |  |  |  |  |  |  |
| N. | **Moser** |  |  |  |  |  |  |  |  |  |  |  |  |  |  |
| N. | **Mursi** |  |  |  |  |  |  |  |  |  |  |  |  |  |  |
| P. | **Georgiou** |  |  |  |  |  |  |  |  |  |  |  |  |  |  |
| P.M. | **Hamade** |  |  |  |  |  |  |  |  |  |  |  |  |  |  |
| S. | **Duodu** |  |  |  |  |  |  |  |  |  |  |  |  |  |  |
| S. | **Gamil** |  |  |  |  |  |  |  |  |  |  |  |  |  |  |
| S. | **Yeung** |  |  |  |  |  |  |  |  |  |  |  |  |  |  |
| T. | **Porat** |  |  |  |  |  |  |  |  |  |  |  |  |  |  |
| U. | **D'Alessandro** |  |  |  |  |  |  |  |  |  |  |  |  |  |  |
| W.L. | **Baxter** |  |  |  |  |  |  |  |  |  |  |  |  |  |  |
| X-L. | **Palmer** |  |  |  |  |  |  |  |  |  |  |  |  |  |  |
